# Quality of life measurement in community-based aged care – understanding variation between clients and between care service providers

**DOI:** 10.1186/s12877-021-02254-2

**Published:** 2021-06-28

**Authors:** Joyce Siette, Mikaela L. Jorgensen, Andrew Georgiou, Laura Dodds, Tom McClean, Johanna I. Westbrook

**Affiliations:** 1 Centre for Health Systems and Safety Research, Australian Institute of Health Innovation, Macquarie University, Macquarie Park, New South Wales 2109 Australia; 2 Centre for Ageing, Cognition and Wellbeing, Macquarie University, Macquarie Park, New South Wales 2109 Australia; 3 Uniting, Sydney, New South Wales 2000 Australia

**Keywords:** Home and community care, Aged care services, Quality indicators

## Abstract

**Background:**

Measuring person-centred outcomes and using this information to improve service delivery is a challenge for many care providers. We aimed to identify predictors of QoL among older adults receiving community-based aged care services and examine variation across different community care service outlets.

**Methods:**

A retrospective sample of 1141 Australians aged ≥60 years receiving community-based care services from a large service provider within 19 service outlets. Clients’ QoL was captured using the ICEpop CAPability Index. QoL scores and predictors of QoL (i.e. sociodemographic, social participation and service use) were extracted from clients’ electronic records and examined using multivariable regression. Funnel plots were used to examine variation in risk-adjusted QoL scores across service outlets.

**Results:**

Mean age was 81.5 years (SD = 8) and 75.5% were women. Clients had a mean QoL score of 0.81 (range 0–1, SD = 0.15). After accounting for other factors, being older (*p* < 0.01), having lower-level care needs (*p* < 0.01), receiving services which met needs for assistance with activities of daily living (*p* < 0.01), and having higher levels of social participation (*p* < 0.001) were associated with higher QoL scores. Of the 19 service outlets, 21% (*n* = 4) had lower mean risk-adjusted QoL scores than expected (< 95% control limits) and 16% (*n* = 3) had higher mean scores than expected.

**Conclusion:**

Using QoL as an indicator to compare care quality may be feasible, with appropriate risk adjustment. Implementing QoL tools allows providers to measure and monitor their performance and service outcomes, as well as identify clients with poor quality of life who may need extra support.

**Trial registration:**

Australian and New Zealand clinical trial registry number: ACTRN12617001212347. Registered 18/08/2017.

**Supplementary Information:**

The online version contains supplementary material available at 10.1186/s12877-021-02254-2.

## Background

Enabling older adults to live independently in the community is a growing priority for health and aged care systems worldwide. Governments want their populations to “age well”, which requires not only an absence of disease and illness but also maintaining a good quality of life (QoL) [1]. Policy makers are therefore increasingly interested in promoting and measuring QoL, which is a dynamic, multi-faceted concept with social and psychological dimensions [1].

Older adults report QoL as a central goal for aged care, with 93% of Australians [2] agreeing that it should be mandatory for providers to publicly report on QoL measures. Quality of life is also considered by older Australians as a valuable indicator for comparing aged care services [3]. While the measurement of quality of life is not currently a mandatory requirement for aged care services, the recent Royal Commission into Aged Care Quality and Safety has emphasized that aged care services should be designed to promote the maintenance of older adults’ QoL by meeting standards in health, safety and personal care [4]. Yet research in both community-based and institutionalized aged care settings has predominantly focused on clinical indicators of care quality, such as incidence of pressure ulcers or number of falls and fall-related fractures [4], providing a limited reflection of the factors that matter most to individuals’ lives beyond basic medical and physical needs [5].

European aged care providers have regularly used social-care related QoL measures since 2009 [5–8]. In the UK, QoL is considered among the top three most useful indicators of care home quality [3]. However, providers are opposed to the publication of individual provider performance data [6] and relatively little information about the quality of individual care homes and provider performance has been made available to the public [7]. Most studies of older adults in the community [9] and institutionalized care [10] exploring QoL have been qualitative, using small, exploratory samples that limit generalizability. Despite known factors that predict QoL for older adults in the wider community (e.g., increased functional status [11, 12], fewer chronic conditions [13], lower levels of depression [14], high-quality social contacts [15]), few studies have examined sociodemographic and service-level predictors of QoL in older adults receiving community-based aged care services [16–18].

Routinely collected data within electronic aged care records and administrative information systems offer a potential way to consistently measure and monitor clinical care quality and social outcomes [19]. By linking sociodemographic and health information from other parts of the information system (i.e., individual-level factors outside the provider’s control that are known to impact QoL), a meaningful comparison between providers can be made to support benchmarking and drive improvements in care [20]. This information can also be used to identify provider-level factors associated with better outcomes and monitor variation in outcomes over time between providers, which is central for informing and supporting increased choice and control for older adults and their families.

This study investigated an Australian cohort where measures of QoL were incorporated into routine assessments of older adults and integrated into their electronic client records in community aged care [21]. This study aimed to identify predictors of QoL among older adults receiving community-based aged care services and examine variation across different community care services.

## Method

### Study population

The study population comprised 1141 people aged 60 years and older who received home and community-based services from Uniting, a non-profit organisation and the largest provider of community aged care services in New South Wales and the Australian Capital Territory in Australia. Clients were included if they completed a QoL assessment together with their case manager as part of their usual care during the period 1 March 2016 to 31 December 2018 [21]. Assessments were completed face to face with clients by staff, typically on an annual basis with the aim to use the information gained to inform care planning. During the study period, 49% of the total client base receiving Commonwealth Home Support Programme services (see definition below) was assessed. Clients who did and did not complete the assessments were similar in terms of age and country of birth, but a greater proportion of women (77% vs 64%) and those receiving a public pension (86% vs 79%) completed the QoL assessment.

### Data source

This study was part of the Ageing Well Project (see protocol [22]), which aimed to determine levels and predictors of social participation and QoL among older adults using community care services. Non-identifiable client data were retrospectively extracted from the provider’s centralised client management system, Carelink+ [23], which included information on community care service use, individual functional needs, client demographics and type of aged care funding received. Measures of social participation and QoL were also recorded in Carelink+ [21, 22].

### Study outcomes

The primary outcome QoL was measured using the ICEpop CAPability Index (ICECAP-O [17]), an instrument developed using rigorous qualitative and quantitative methods and validated widely [17, 18]. The ICECAP-O instrument measures capability by assessing an individual’s capacity to perform certain actions and achieve certain states [17]. ICECAP-O measures five dimensions (attachment, security, role, enjoyment, and control), with one question per dimension and four available responses. For example, the dimension attachment has the following four statements in which an individual is asked to select the one that best matches their response: “I can have all of the love and friendship that I want”, “I can have a lot of the love and friendship that I want”, “I can have a little of the love and friendship that I want”, or “I cannot have any of the love and friendship that I want”. A final score between 0 and 1 is computed, with a higher score indicating higher QoL. Specifically, a value of 0.556 is equivalent to having slight capability in all dimensions and 0.866 indicates having a lot of capability in all dimensions [24].

### Predictors of QoL

The selection of factors that may predict QoL was guided by previous literature including the Engel’s Comprehensive Model [25] and the physical-environment model [26, 27]. These models suggest that predisposing factors, social factors, and personal need factors may be crucial to QoL in older adults. All factors were obtained from clients’ electronic records. Potential predisposing factors included demographic variables such as age, gender, country of birth, language spoken, pension status, geographic location, and socioeconomic status. The client’s suburb of residence was used to classify remoteness based on the Accessibility/Remoteness Index of Australia (ARIA) [28]. Socioeconomic status was calculated using the index of relative socio-economic advantage and disadvantage (IRSAD) based on the client’s suburb or postcode [29].

Social factors included marital status and social participation levels, measured using the Australian Community Participation Questionnaire (ACPQ-SF15 [30]). The ACPQ-SF15 was conducted typically at the same time as the ICECAP-O and consists of 14 items to assess engagement with the community, including contact with immediate household, extended family, friends, and neighbours, and participating in organised community activities, among others. It yields a single total score ranging from 0 to 7 with lower scores denoting less community participation.

Service factors included service provision, funding type, and data from the Australian Community Care Needs Assessment (ACCNA), an annual screening tool assessment to determine funding eligibility for home care services [31]. The ACCNA includes information about a client’s levels of dependency in (i) five basic activities of daily living (ADLs) (i.e., bathing, dressing, eating, walking and toileting), (ii) five instrumental activities of daily living (IADLs) (i.e., housework, getting places, shopping, taking medicine, and handling money). The number of unmet needs for assistance with ADLs and IADLs were determined using data from the ACCNA and service use data (i.e., where a client requires assistance with one or more ADLs or IADLs, but no matching services were provided to meet those needs). Refer to Additional file 1 for further detail.

Services provided to community care clients included personal care (e.g., showering assistance), support services (e.g., in-home social calls), nursing (e.g., medication management) and other allied health or clinical services (e.g., providing mobility aids). The start time, end time, date, and service type for each occasion of service was extracted from client records. We examined service provision factors which included the median number of service hours received per week, the type of services received, and the total number of different service types received for up to 91 days prior to the QoL assessment. Please refer to Additional file 1 for more information.

There are two main government-subsidised community aged care programs in Australia. The Home Care Package (HCP) program provides coordinated care packages for people with higher level and more complex care needs to enable them to live independently in their own homes, and typically includes multiple service types such as domestic assistance and clinical care. The other main form of care is the Commonwealth Home Support Programme (CHSP) which provides entry-level support for basic assistance needs. In Australia, client services are delivered or coordinated from a base known as a ‘service outlet’. A service provider may have many outlets in different geographical locations or just one. An external government accreditation agency undertakes a quality review of each service outlet every 3 years [32]. The service outlet responsible for providing services for each client was determined based on the date of the QoL assessment.

### Statistical analyses

#### Client predictors of QoL

Univariate analyses were first performed to screen for potential predictors of QoL based on previous literature [25–27]. Variables that were statistically significant at the *p* < 0.2 level in the univariate analyses were included in multiple regression models. Multiple linear regression analyses, using forced entry, were used to examine predictors of QoL. In the first step, all predictors were entered in one block according to their effect sizes. In step two, predictors were entered one by one in order of their standardized beta coefficients (ßs) in the previous analysis as long as they contributed to the model (i.e., significant ΔR^2^ increase). Regression diagnostics were performed to investigate any violation of the assumptions of normality, linearity, multicollinearity and homoscedasticity (i.e., checked by examination of intercorrelations, tolerances and variance inflation factors (VIF)). The models were also examined by visual inspection of the distributions and normal probability plots of their standardized residuals. Level of statistical significance was set at 0.05. Statistical analyses were performed with IBM SPSS Statistics V25. A sample size calculation was calculated and reported in our protocol paper [22].

#### Variation in QoL between service outlets

Funnel plots were used to examine variation in mean QoL scores between service outlets [33]. Funnel plots allow for the identification of outlying performers while accounting for less reliable estimates from smaller service outlets, avoiding spurious ‘league table’ rank ordering based on point estimates alone [33]. For this part of the analysis, only QoL assessments from 1 July 2017 were used. Prior to this time, only one service outlet was using the ICECAP-O instrument [22]. Service outlets who assessed less than 10 clients were also excluded (*n* = 2 outlets).

Mean scores for each service outlet were adjusted for case-mix using multiple linear regression models. Factors included in the case-mix adjusted models were age, gender, socioeconomic status, and care needs. These factors represent consistently reported predictors of QoL among older adults [11, 12, 15, 34, 35] that are not within the control of the provider. Each service outlet’s expected score was obtained by taking the mean score of their clients’ predicted values from the model, given their covariate values. The observed mean score was divided by the expected mean score and then multiplied by the study population mean score to obtain the risk-adjusted mean QoL score for each service outlet [36].

In the funnel plots, risk-adjusted scores for each service outlet were plotted against the number of clients completing the ICECAP-O tool. For each quarter, 95 and 99.8% ‘control limits’ were calculated based on the mean and standard deviation of the study population’s QoL scores. Service outlets with rates outside of these limits were considered outliers.

## Results

The characteristics of the 1141 clients receiving community care services are described in Table 1. Mean age was 81.5 years and the majority of clients were aged 80 to 89 years old (41.2%). Most clients were female (75.5%), living in a major city (79.1%), born in a country where the main language is English (59.9%) and were receiving entry-level support (80.1%). They received an average of one service type per week with a mean number of five service hours per week. The most common service was attendance at a Day Centre (63.4%). Half the client sample had no need for assistance with ADLs (e.g., bathing, dressing) (50.3%) and a third had no need for assistance with IADLs (e.g., housework, shopping) (33.7%). Most clients had their needs met by the provider (46.2%). Clients had a mean ICECAP-O score of 0.81 (range 0–1, SD = 0.15).

**Table 1:** Demographics and client characteristics of 1141 older adults receiving aged care services

Characteristic	n (%)
Gender	
Female	862 (75.5)
Male	279 (24.5)
Missing	4 (0.4)
Age	
Mean [SD]	81.5 [7.9]
60–69	62 (6.0)
70–79	368 (35.6)
80–89	426 (41.2)
≥ 90 years	179 (17.3)
Relationship status	
Living with someone	388 (34.0)
Living alone	506 (44.3)
Missing	247 (21.6)
Country of birth	
English speaking country	684 (59.9)
Non-English speaking country	457 (40.1)
Remoteness^a^	
Major city	902 (79.1)
Inner regional	157 (13.8)
Outer regional/remote	82 (7.2)
Socio-economic status	
1 (lowest)	119 (10.4)
2	134 (11.7)
3	79 (6.9)
4	57 (5.0)
5	57 (5.0)
6	97 (8.5)
7	36 (3.2)
8	76 (6.4)
9	107 (9.4)
10 (highest)	383 (33.5)
Pension status	
Pension	980 (85.9)
No pension	86 (7.5)
Missing	75 (6.6)
Funding package^b^	
Entry-level care	914 (80.1)
More complex care	198 (17.4)
Private/Veterans	7 (0.7)
Services	
ean number of services [SD]	1.37 [0.9]
Mean service hours per week [SD]	5.1 [3.9]
Used service^c^	
Day Centre	660 (63.8)
Domestic Assistance	131 (12.7)
Social Support	84 (8.1)
Personal Care	54 (5.2)
Shopping	53 (5.1)
Respite Care	39 (3.8)
Meal Preparation	28 (2.7)
Outings	16 (1.5)
Allied Health Therapy	9 (0.9)
Medication Assistance	8 (0.8)
Service Type Cluster^d^	
Day Centre	844 (81.5)
Social Support	151 (14.6)
Outings	40 (3.9)
Number of ADLs needing help^e^	
0	521 (50.3)
1	37 (3.6)
2	14 (1.4)
3	5 (0.05)
4	4 (0.04)
5	4 (0.4)
Missing	450 (43.5)
Number of IADLs needing help^f^	
0	349 (33.7)
1	93 (9.0)
2	57 (5.5)
3	55 (5.3)
4	53 (3.2)
5	17 (1.6)
Missing	431 (41.6)
Number of unmet needs^g^	
0	157 (46.2)
1	53 (15.6)
2	24 (7.1)
3	25 (7.4)
4	8 (2.4)
5	1 (0.3)
Missing	72 (21.2)
Quality of life [SD]	0.81 [0.15]
Social Participation [SD]	3.58 [1.87]

^a^Accessibility/Remoteness Index of Australia and Index of Relative Socio-economic Advantage and Disadvantage based on each person’s suburb or postcode if no matched suburb. ^b^More complex care defined by clients receiving Home Care Package, and entry-level care defined by clients receiving Commonwealth Home Support Package (CHSP). ^c^Ten most commonly used services only; other services used were nursing services, assessment, coordination and advocacy. ^d^A profile of service types used by each cluster can be found in Additional file 1. ^e^ADLs consisted of bathing, dressing, eating, walking and toileting. Number refers to the number of assistance with ADLs ^f^IADLs consisted of doing housework, getting backs, shopping, taking medicine, and handling money. Number refers to the number of needs of assistance with IADLs. ^g^Numbers and percentage presented for the category “Has need and not provided by service” only

### Client predictors of QoL

Univariate associations between QoL scores and predisposing, personal needs and social factors are shown in Table 2. After accounting for other factors in the final multiple linear regression model, being older, living in regional or remote areas, having lower-level care needs, higher levels of social participation, and receiving services which met a greater number of needs for assistance with functional and instrumental activities of daily living were associated with significantly higher QoL (Table 3). Combined, these variables explained 21% of the variance in QoL scores for older adults. High levels of social participation and having services that met needs for assistance with activities of daily living were found to be key predicting factors for QOL.

**Table 2:** Univariate analyses of associated factors for quality of life in older adults in community care

Variable	Subgroup	Quality of life mean (SD)	t/F^a^	*P*
Gender	Female	0.82 (0.14)	1.195	0.031*
	Male	0.78 (0.17)		
Age	60–69	0.82 (0.18)	1.354	0.001*
	70–79	0.82 (0.14)		
	80–89	0.81 (0.16)		
	≥ 90 years	0.78 (0.15)		
Relationship status	Living with someone	0.81 (0.15)	1.357	0.001*
	Living alone	0.81 (0.15)		
Country of birth	English speaking country	0.82 (0.15)	0.96	0.654
	Non-English speaking country	0.81 (0.16)		
Remoteness	Major city	0.81 (0.16)	1.218	0.019*
	Inner regional	0.83 (0.12)		
	Outer regional/remote	0.79 (0.15)		
Socio-economic status	Lowest quintile	0.82 (0.15)	0.924	0.786
	Not in lowest quintile	0.81 (0.15)		
Pension status	Pension	0.81 (0.15)	1.222	0.02*
	No pension	0.79 (0.18)		
Funding package	Entry-level care	0.83 (0.14)	1.379	< 0.001*
	More complex care	0.73 (0.17)		
Service type cluster	Mostly Day Centre	0.83 (0.14)	1.403	< 0.001*
	Mostly Social Support	0.74 (0.18)		
	Mostly Outings	0.77 (0.17)		
Services	Above mean service hours	0.82 (0.15)	1.172	0.06
	Below mean service hours	0.80 (0.15)		
Number of ADLs needing help^a^	0	0.82 (0.15)	3.915	< 0.001*
	1	0.72 (0.14)		
	2	0.59 (0.23)		
	3	0.56 (0.13)		
	4	0.66 (0.08)		
	5	0.68 (0.19)		
Number of IADLs needing help^b^	0	0.83 (0.13)	1.736	< 0.001*
	1	0.74 (0.19)		
	2	0.76 (0.17)		
	3	0.78 (0.16)		
	4	0.75 (0.19)		
	5	0.78 (0.14)		
Number of unmet needs	0	0.83	7.27	< 0.001*
	1	0.74		
	2	0.76		
	3	0.72		
	4	0.92		
	5	0.85		
Social Participation	Household - Below the mean	0.80 (0.16)	1.48	< 0.001*
	- Above the mean	0.82 (0.15)		
	Family - Below the mean	0.77 (0.17)	1.541	< 0.001*
	- Above the mean	0.84 (0.13)		
	Friends - Below the mean	0.76 (0.17)	1.701	< 0.001*
	- Above the mean	0.86 (0.12)		
	Neighbours - Below the mean	0.77 (0.17)	1.457	< 0.001*
	- Above the mean	0.85 (0.13)		
	Religious - Below the mean	0.79 (0.17)	1.014	0.438
	- Above the mean	0.84 (0.12)		
	Community- Below the mean	0.75 (0.18)	1.753	< 0.001*
	- Above the mean	0.86 (0.11)		
	Affairs - Below the mean	0.77 (0.17)	1.428	< 0.001*
	- Above the mean	0.86 (0.11)		
Social participation total score	0	0.64 (0.22)	2.086	< 0.001*
	1	0.72 (0.15)		
	2	0.76 (0.16)		
	3	0.80 (0.14)		
	4	0.85 (0.12)		
	5	0.87 (0.10)		
	6	0.89 (0.10)		
	7	0.91 (0.08)		

^a^ADLs consisted of bathing, dressing, eating, walking and toileting. Number refers to the numbers of needs for assistance with ADLs. ^b^IADLs consisted of doing housework, getting backs, shopping, taking medicine, and handling money. Number refers to the numbers of assistance with IADLs

*refers to significant *p*-value

**Table 3:** Summary of multiple linear regression analyses for predictors of quality of life in 925 older community care adults

Predictors	Quality of life (*N* = 925)				
	B	SE B	ß	***p***-value	95% CI
Constant	.694	.037		.000	.662, .766
Age	.027	.011	.076	.014*	.005, .049
Gender	.006	.005	.035	.261	−.005, .017
Relationship status	.020	.011	.057	.076	−.002, .043
Remoteness	−.025	.009	−.087	.004**	−.042, −.008
Funding Type	−.042	.016	−.106	.009**	−.073, −.01
Service hours	−.010	.010	−.031	.306	−.029, .009
Service type	−.003	.012	−.012	.767	−.027, .020
ADLs needs met	−.033	.010	−.107	.001***	−.053, −.013
IADLs needs met	−.006	.005	−.039	.247	−.016, .004
Social participation	.034	.003	.386	< 0.001***	.029, .040
		R^2^	0.21		
		F	22.12		

B, unstandardized coefficients; SE B, unstandardized coefficient standard error; ß, standardized coefficients beta; 95% CI = confidence interval for B

*p < 0.05; **p < 0.01; ***p < 0.001

### Variation in QoL between service outlets

**Fig. 1:**
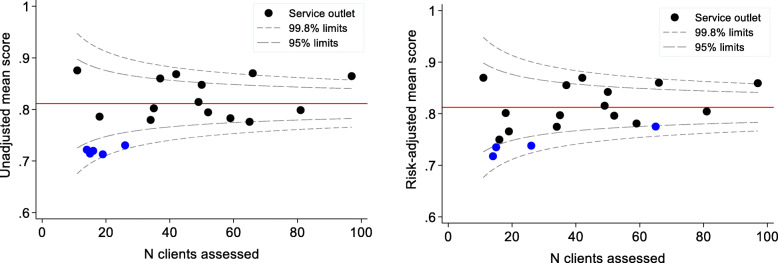
Unadjusted (left) and risk-adjusted (right) mean QoL scores comparing 19 community care service outlets

A total of 19 service outlets undertook QoL assessments with an average of 41 clients each over the study period (SD = 24, max 97 clients). Unadjusted and risk-adjusted mean QoL scores by service outlet are presented in Fig. 1 using funnel plots. Of the 19 service outlets, four (21%) had lower mean scores than would be expected after accounting for case-mix (below 95% control limits). A further three service outlets (16%) had higher mean scores than would be expected (above 95% control limits). Nearly two-thirds of outlets performed within the expected range (63.1%). Outlets with higher mean scores than would be expected provided services to a greater number of clients than outlets whose scores were within or below control limits (mean 68 vs 36 clients, t(17) = − 2.32).

## Discussion

Our study describes the QoL of a large sample of people in the community who receive aged care services. We found that QoL varied between clients as well as between different community care service outlets. The findings identified factors that predicted QoL, including that social participation opportunities and provision of care services that meet older adults’ needs are associated with higher QoL. We further showed that validated QoL measures can feasibly be integrated into routine assessments and when stored electronically, permit risk-adjusted analyses to allow benchmarking between service providers.

Addressing the unmet care and support needs of an ageing population, and designing services and solutions centred around what older people need and want, remains an urgent public health priority [37]. Previous large scale studies indicating that the level, maintenance and development of high quality social support networks contribute to positive QoL [38–42]. This is because social engagement can increase an individual’s social networks, which leads to attachment, social approval, belonginess, social identity and increased access to social support structures [43]. In particular, older adults’ social networks could provide access to functional support and assistance from family members, neighbours, friends and service providers. This type of support is critical and is related to older adults’ perception of control and improved wellbeing [41]. Our results indicate that moderate levels of social participation with family, friends, neighbours and community is evident in our sample. We further highlight the vital role of social participation for older adults receiving aged care services in the community, whereby interpersonal interactions and engagement in community-based activities through care service provision is a valuable influence on overall QoL.

We found that older age was a predictor of better QoL, which replicates previous findings of a U-shaped association between age and wellbeing with the lowest occurring in middle age and higher wellbeing in younger and older adults [44, 45]. This result is often explained by the socio-emotional selectivity theory [46] which argues that with advancing age, individuals accumulate emotional wisdom that leads to the selection of more emotionally satisfying events, friendships, and experiences. Despite changing circumstances such as the death of loved ones, loss of status associated with retirement, deteriorating health and reduced income, older adults often maintain and even increase self-reported wellbeing by focusing on a more limited set of social contacts and experiences [46].

Locality was found to influence QoL. Our results support previous findings that older adults living in rural or regional areas are more likely to have better life satisfaction and QoL [47, 48]. In line with recent Australian household-based studies (e.g., HILDA), rural individuals expressed greater satisfaction with relationships compared to urban populations. However, the association between locality and QoL remains complex (e.g., [49]) and can be influenced by a range of factors including presence of existing social networks, healthcare availability, level of educational attainment, economic opportunities, environmental conditions, and area accessibility [50]. An understanding of the relative contribution of these factors towards QoL would be helpful in shaping future policies and interventions, particularly for rural communities, but unfortunately, such data were not collected in this study.

Contrary to our initial expectations, our findings showed a relatively high QoL for community care older adults, compared to national and international samples. Clients’ QoL scores in this study are similar to older community-dwelling adults in both Australia [18] and the UK [17, 24], but higher than social care users in the UK [16] and clinical post-acute rehabilitation outpatients in Australia [18]. This contrasts with earlier research that has shown that aged care services are associated with a negligible impact on outcomes [51, 52]. These findings might be at least partly attributable to the longer time for which clients in the present study had been receiving community care services (> 12 months), which may have supported clients to develop meaningful relationships with staff and other clients, allowing them to reap the full benefit of care services. Another explanation for the higher than expected QoL in our study is that a large proportion of our cohort had only low level care needs, and therefore may be comparable to the wider general population of older adults.

We found that client QoL scores were higher in outlets with the largest number of clients, with a large proportion of all clients accessing day centre services. As social interaction is often the core element of day centre services [53], outlets that are servicing larger client populations may be able to provide new and wider social networks. For many older adults, family and close friends can provide companionship and facilitate social and pleasurable activities, which are critical to maintaining QoL in older age [54]. Aged care providers can build on these relationships to improve clients’ QoL by tailoring community-based activities and services to build and sustain social networks for individuals with lower levels of social participation as well as using alternative methods, such as providing access to digital technology to enhance social connectedness [55].

Our results show that meeting the needs of older adults through providing services that support activities of daily living was associated with higher QoL. To our knowledge, this is the first study to demonstrate this relationship. Previous qualitative studies of older adults or family carers [37, 52, 56] have identified the impact of coordination of services, access to information and preventative strategies on clinical (e.g., physical function, medication management) and QoL outcomes. In Australia, due to maximum funding budgets for government-subsidised home care [57], individuals with higher needs may not be as well supported because of the limited number of available hours to address their needs. Further research is required to identify how much and what type of home care services can successfully support older adults to live independently. This could include tailored identification of specific types of assistance and reablement that are important to each client’s QoL. A systematic review comparing different care models of non-medical home and community services for older persons found that different models (e.g., integrated care, consumer-directed care) impacted on different outcomes (physical health, ADLs, quality of life), and concluded that a focus should be on combining the successful features (e.g., case management, integrated care, consumer directed care) to maximise outcomes [52]. Understanding how different models of service and care provision are effective in enhancing QoL among clients, as well as an understanding of the relative priorities of needs for individual clients, would be beneficial in tailoring future care plans.

Our previous qualitative research has highlighted that entering into discussions about social needs and QoL with a staff member facilitates better matching of client needs to appropriate services [21]. Other evidence also suggests that older adults with lower QoL may benefit more from programs that involve iterative feedback from a healthcare professional and are tailored to their specific social, cognitive and physical needs [58]. However, discussions about QoL can be emotionally difficult for staff [21] and more resource intensive than traditional clinical process indicators. This may result in variation between outlets in whether staff use QoL assessment simply to obtain a score, or use it as a basis for a conversation to match services with client’s needs. Regardless, our experiencing has indicated that staff find such conversations valuable [21].

Quality indicators need to be sensitive, reliable, evidence-based and be able to discriminate between care providers [59]. The variation we found in QoL between different outlets after adjusting for key client factors demonstrates that QoL measures have the potential to be used as a meaningful quality indicator in community aged care. We found that 21% of service outlet centres had lower risk-adjusted mean scores than would have been expected given their client population and 16% better than expected. This variation in QoL may reflect differences between outlets in the availability and types of services they offer that can meet clients’ needs and preferences. Through benchmarking these data, providers may be better able to assess which of their service outlets and client populations needs more support in these areas, and which outlets are performing well and can be champions.

Utilising data obtained from aged care electronic management systems for measuring outcomes has several benefits. Firstly, these different systems enable linkage between sociodemographic and QoL information to risk adjust and compare provider performance. Secondly, they enable providers to examine what services they are supplying to whom and evaluate whether such services are associated with improvements in client QoL. Finally, using already established organisational processes (e.g., forms embedded in client electronic systems, staff awareness and training for QoL assessments) [21] to support QoL measurement among clients can support the regulation of aged care standards. The next stage is to ensure QoL assessments are carried out by aged care staff systematically using QoL measures that have been developed specifically for aged care settings [60].

### Limitations

This study only examined clients who had completed QoL tools at a single point in time. Future work should include a wider client sample and evaluate the impact of services on changes in client’s QoL scores over time. Furthermore, while we adjusted for essential factors such as socioeconomic status, age and care needs, fully disentangling the impact of the service provider on QoL compared to the local environment that clients live in requires additional area-level information that was not available in this study.

## Conclusion

There is a growing interest in the measurement of quality to support transparency in the aged care sector and to improve services. QoL, a promising care quality indicator, has the potential to be used to identify variation in service performance and be used by providers and governments to benchmark community-based aged care quality.

## Supplementary Information

**Additional file 1: Supplementary Table 1.** Predictor factors included in analyses and their description. **Supplementary Table 2.** Median Service Hours (IQR) used per week for each service type cluster.

## Data Availability

The data that support the findings of this study are available from Uniting but restrictions apply to the availability of these data, which were used under a memorandum of understanding for the current study, and so are not publicly available.

## References

[1] World Health Organization (2002). Active ageing: A policy framework.

[2] Yates I, Irlam C (2018). Measuring quality and consumer choice in aged care.

[3] Kumpunen S, Trigg L, Holder J (2019). Helping older people to use quality information to choose residential care. Helping older people to use quality information to choose residential care.

[4] Briggs L, Tracey R (2019). Royal Commission into Aged Care Quality and Safety, Interim Report: Neglect.

[5] Arling G, Kane RL, Lewis T, Mueller C (2005). Future development of nursing home quality indicators. Gerontologist.

[6] House of Commons and Health Committee (2013). 2012 accountability hearing with the Care Quality Commission in Seventh Report of Session 2012–13.

[7] Malley J, Maresso A, Leone T, Mor V (2014). Regulating the quality and safety of long-term care in England, in Regulating Long-Term Care Quality: An International Comparison.

[8] OECD (2013). OECD Guidelines on Measuring Subjective Well-being.

[9] Cardona B (2018). Measuring outcomes of community aged care programs: challenges, opportunities and the Australian community outcomes measurement ACCOM tool. Health Qual Life Outcomes.

[10] Bergland A, Narum I (2007). Quality of life demands comprehension and further exploration. J Aging Health.

[11] Netuveli G, Wiggins RD, Hildon Z, Montgomery SM, Blane D (2006). Quality of life at older ages: evidence from the English longitudinal study of aging (wave 1). J Epidemiol Community Health.

[12] Davis JC, Bryan S, Best JR, Li LC, Hsu CL, Gomez C, Vertes KA, Liu-Ambrose T (2015). Mobility predicts change in older adults’ health-related quality of life: evidence from a Vancouver falls prevention prospective cohort study. Health Qual Life Outcomes.

[13] Chan SW-C (2009). Predictors of change in health-related quality of life among older people with depression: a longitudinal study. Int Psychogeriatr.

[14] Stone AA, Schwartz JE, Broderick JE, Deaton A (2010). A snapshot of the age distribution of psychological well-being in the United States. Proc Natl Acad Sci.

[15] Pinquart M, Sörensen S (2000). Influences of socioeconomic status, social network, and competence on subjective well-being in later life: a meta-analysis. Psychol Aging.

[16] Hackert MQN, Exel J, Brouwer WBF (2017). Valid outcome measures in care for older people: comparing the ASCOT and the ICECAP-O. Value Health.

[17] Flynn TN, Chan P, Coast J, Peters TJ (2011). Assessing quality of life among British older people using the ICEPOP CAPability (ICECAP-O) measure. Appl Health Econ Health Policy.

[18] Couzner L, Ratcliffe J, Crotty M (2012). The relationship between quality of life, health and care transition: an empirical comparison in an older post-acute population. Health Qual Life Outcomes.

[19] Douglas HE (2017). Implementing information and communication technology to support community aged care service integration: lessons from an Australian aged care provider. Int J Integr Care.

[20] Burkett E, Martin-Khan MG, Gray LC (2017). Quality indicators in the care of older persons in the emergency department: a systematic review of the literature. Aust J Ageing.

[21] Siette J, Georgiou A, Jorgensen M, O'Donnell C, Westbrook J (2018). Integrating social engagement instruments into Australian community aged care assessments to enhance service provision. Health Soc Care Commun.

[22] Brett L, Georgiou A, Jorgensen M, Siette J, Scott G, Gow E, Luckett G, Westbrook J (2019). Ageing well: evaluation of social participation and quality of life tools to enhance community aged care (study protocol). BMC Geriatr.

[23] Carelink+. Carelink+ Community Care Software and Solutions. 2019; Available from: https://carelinkplus.com.au/.

[24] Coast J, Flynn TN, Natarajan L, Sproston K, Lewis J, Louviere JJ, et al. Valuing the ICECAP capability index for older people. Soc Sci Med. 2008;67(5):874–82. 10.1016/j.socscimed.2008.05.015. Accessed 12 Feb 2021. 10.1016/j.socscimed.2008.05.015 18572295

[25] Engel GL (1978). The biopsychosocial model and the education of health professionals. Ann N Y Acad Sci.

[26] Andersen RM. Revisiting the behavioral model and access to medical care: does it matter? J Health Soc Behav. 1995;36(1):1-10. 7738325

[27] Sallis JF, Owen N, Fisher E (2015). Ecological models of health behavior. Health Behav.

[28] Wenze SJ, Miller IW (2010). Use of ecological momentary assessment in mood disorders research. Clin Psychol Rev.

[29] SA1 SEIFA (2011). 2011 - the index of relative socio-economic advantage and disadvantage (IRSAD).

[30] Berry HL, Rodgers B, Dear KB (2007). Preliminary development and validation of an Australian community participation questionnaire: types of participation and associations with distress in a coastal community. Soc Sci Med.

[31] Samsa P (2007). The Australian community care needs assessment (ACCNA): towards a national standard.

[32] Australian Commission on Safety and Quality in Health Care (2019). Australian health service safety and quality accreditation scheme.

[33] Rakow T, Wright RJ, Spiegelhalter DJ, Bull C (2015). The pros and cons of funnel plots as an aid to risk communication and patient decision making. Br J Psychol.

[34] Brett CE, Gow AJ, Corley J, Pattie A, Starr JM, Deary IJ (2012). Psychosocial factors and health as determinants of quality of life in community-dwelling older adults. Qual Life Res.

[35] Inglehart R (2002). Gender, aging, and subjective well-being. Int J Comp Sociol.

[36] Iezzoni LI (1995). Risk adjustment for medical effectiveness research: an overview of conceptual and methodological considerations. J Invest Med.

[37] Abdi S, Spann A, Borilovic J, de Witte L, Hawley M (2020). Correction to: understanding the care and support needs of older people: a scoping review and categorisation using the WHO international classification of functioning, disability and health framework (ICF). BMC Geriatr.

[38] Bowling A (2010). Do older and younger people differ in their reported well-being? A national survey of adults in Britain. Fam Pract.

[39] Leung BW-C, Moneta GB, McBride-Chang C (2005). Think positively and feel positively: optimism and life satisfaction in late life. Int J Aging Hum Dev.

[40] Lei P, Xu L, Nwaru BI, Long Q, Wu Z (2016). Social networks and health-related quality of life among Chinese old adults in urban areas: results from 4th National Household Health Survey. Public Health.

[41] Rafnsson SB, Shankar A, Steptoe A (2015). Longitudinal influences of social network characteristics on subjective well-being of older adults: findings from the ELSA study. J Aging Health.

[42] Litwin H, Shiovitz-Ezra S (2011). Social network type and subjective well-being in a national sample of older Americans. Gerontologist.

[43] Takagi D, Kondo K, Kawachi I (2013). Social participation and mental health: moderating effects of gender, social role and rurality. BMC Public Health.

[44] Steptoe A, Deaton A, Stone AA (2015). Subjective wellbeing, health, and ageing. Lancet (London, England).

[45] Szabó Á, Hyde M, Towers A. One slope does not fit all: longitudinal trajectories of quality of life in older adulthood. Qual Life Res. 2021. 10.1007/s11136-021-02827-z. 10.1007/s11136-021-02827-z 33843014

[46] Carstensen LL, Fung HH, Charles ST (2003). Socioemotional selectivity theory and the regulation of emotion in the second half of life. Motiv Emot.

[47] Hogan A (2008). The social wellbeing of rural Australians: an analysis of the household, income and labour dynamics in Australia (HILDA) longitudinal dataset.

[48] Carta MG, Aguglia E, Caraci F, Dell'Osso L, di Sciascio G, Drago F, del Giudice E, Faravelli C, Hardoy MC, Lecca ME, Moro MF, Calò S, Casacchia M, Angermeyer M, Balestrieri M (2012). Quality of life and urban / rural living: preliminary results of a community survey in Italy. Clin Pract Epidemiol Mental Health.

[49] Tsai SY, Chi LY, Lee LS, Chou P (2004). Health-related quality of life among urban, rural, and island community elderly in Taiwan. J Formos Med Assoc.

[50] Australian Institute of Health and Welfare (2019). Rural & remote health.

[51] Bulamu N, Kaambwa B, Gill L, Cameron I, McKechnie S, Fiebig J, Grady R, Ratcliffe J (2017). Impact of consumer-directed care on quality of life in the community aged care sector. Geriatr Gerontol Int.

[52] Low L-F, Yap M, Brodaty H (2011). A systematic review of different models of home and community care services for older persons. BMC Health Serv Res.

[53] Orellana K, Manthorpe J, Tinker A (2018). Day centres for older people: a systematically conducted scoping review of literature about their benefits, purposes and how they are perceived. Ageing Soc.

[54] van Leeuwen KM, van Loon MS, van Nes FA, Bosmans JE, de Vet HCW, Ket JCF, Widdershoven GAM, Ostelo RWJG (2019). What does quality of life mean to older adults? A thematic synthesis. PLoS One.

[55] Morris ME, Adair B, Ozanne E, Kurowski W, Miller KJ, Pearce AJ, Santamaria N, Long M, Ventura C, Said CM (2014). Smart technologies to enhance social connectedness in older people who live at home. Australas J Ageing.

[56] McGilton KS, Vellani S, Yeung L, Chishtie J, Commisso E, Ploeg J, Andrew MK, Ayala AP, Gray M, Morgan D, Chow AF, Parrott E, Stephens D, Hale L, Keatings M, Walker J, Wodchis WP, Dubé V, McElhaney J, Puts M (2018). Identifying and understanding the health and social care needs of older adults with multiple chronic conditions and their caregivers: a scoping review. BMC Geriatr.

[57] My Aged Care (2021). Home Care Package costs and fees.

[58] Niclasen J (2018). Mental health interventions among older adults: a systematic review. Scand J Public Health.

[59] Mainz J (2003). Defining and classifying clinical indicators for quality improvement. Int J Qual Health Care.

[60] Ratcliffe J, Cameron I, Lancsar E, Walker R, Milte R, Hutchinson CL, Swaffer K, Parker S (2019). Developing a new quality of life instrument with older people for economic evaluation in aged care: study protocol. BMJ Open.

