# Supplementary material for: Quality of life measurement in community-based aged care – understanding variation between clients and between care service providers
Source: BMC Geriatr. 2021 Jun 28;21:390. doi: 10.1186/s12877-021-02254-2 (PMC8240205; doi:10.1186/s12877-021-02254-2)
Supplement: Supplementary file 1 — Additional file 1: Supplementary Table 1. Predictor factors included in analyses and their description. Supplementary Table 2. Median Service Hours (IQR) used per week for each service type cluster. [file 12877_2021_2254_MOESM1_ESM.docx]

**Supplementary Material**

**Supplementary Table 1. Predictor factors included in analyses and their description.**

| **Demographic Variables** | |
| --- | --- |
| Client Age | Calculated from client’s date of birth. |
| Client Gender | Indicates the client’s gender (as male or female). |
| Client Marital status | A field indicating the marital status of the client. |
| Client Country of birth | A code indicating the country of birth and the language spoken, i.e., Australia, an English-speaking country, a non-English speaking country. |
| Client Language | A code indicating the main language spoken, i.e., English or non-English. |
| Client IRSAD | Index of Relative Socio-economic Advantage and Disadvantage (IRSAD) based on client’s suburb or client postcode where no matched suburb.  Decile within NSW (0 to 10).  The IRSAD is a continuum of disadvantage to advantage and is based on variables relating to a range of factors such as income, employment, education, occupation, internet connection, housing, family structure, marital status and disability. Each area is allocated a score, where a low score indicates relatively greater disadvantage and a lack of advantage; while a high score indicates a relative lack of disadvantage and greater advantage (ABS 2013).  The areas are grouped into deciles (10 categories of advantage/disadvantage, with 1 being the most disadvantaged and 10 being the most advantaged), and quintiles (5 categories of advantage/disadvantage, |
| Client ARIA | Accessibility / Remoteness Index of Australia (2011) based on client’s suburb or client postcode where no matched suburb. |
| Client Pension status | Coded as whether the client is receiving a pension (aged or government or other) or not. |
| **Service Variables** | |
| Funding type | Home Care Package (more complex care), or Commonwealth Home Support Program (entry-level care). |
| Service hours per week | Median number of service hours per week for up to 91 days before. |
| Service days per week | Median number of days that services were delivered per week for up to 91 days before. |
| Most common service | The service type that made up the largest proportion of service hours delivered to the client for up to 91 days before. |
| Number of service types | The number of different service types delivered to the client for up to 91 days before. |
| **Needs Assessment Variable** | |
| ACCNA Functional Toileting | Additional item about function for toileting. Three possible response choices (unable to do alone, some difficulty, no difficulty). |
| ACCNA Functional Moving Around | Additional item about function of getting out of bed and moving around. Three possible response choices (unable to do alone, some difficulty, no difficulty). |
| ACCNA Functional Housework | Can you do your housework? Three possible response choices (unable to do alone, with some help and without help). |
| ACCNA Functional Walking | Can you get to places out of walking distance? Three possible response choices (unable to do alone, with some help and without help). |
| ACCNA Functional Shopping | Can you go out for groceries or clothes (assuming you have transportation)? Three possible response choices (unable to do alone, with some help and without help). |
| ACCNA Functional Medicine | Can you take your own medicine? Three possible response choices (unable to do alone, with some help and without help). |
| ACCNA Functional Money | Can you handle your own money? Three possible response choices (unable to do alone, with some help and without help). |
| ACCNA Functional Walk | Can you walk? Three possible response choices (unable to do alone, with some help and without help). |
| ACCNA Functional Bath | Can you take a bath or shower? Three possible response choices (unable to do alone, with some help and without help). |
| ACCNA Functional Total | Sum of the above functions from Housework to Bath |
| Unmet Personal Care | Are ACCNA needs being met by services provided in the period up to 91 days before. 1 indicate has need and no service provided. 0 has need and service provided OR client does not have need. Personal Care refers to bathing, dressing or toileting assistance. |
| Unmet Domestic | Unmet needs for domestic assistance. Refer to above for codes. |
| Unmet Shopping | Unmet needs for shopping assistance. Refer to above for codes. |
| Unmet Medication | Unmet needs for medication assistance. Refer to above for codes. |
| Unmet Transport | Unmet needs for transport assistance. Refer to above for codes. |
| **Social Variable** | |
| Social Engagement Household | Contact with immediate household. 7 scale response ranging from never (1) to always (7). |
| Social Engagement Family | Contact with extended family. 7 scale response ranging from never (1) to always (7). |
| Social Engagement Friends | Contact with friends. 7 scale response ranging from never (1) to always (7). |
| Social Engagement Neighbours | Contact with neighbours. 7 scale response ranging from never (1) to always (7). |
| Social Engagement Religious Observance | Participation in any religious observances. 7 scale response ranging from never (1) to always (7). |
| Social Engagement Community Activities | Participation in organised community activities. 7 scale response ranging from never (1) to always (7). |
| Social Engagement Affairs | Taking an active interest in current affairs. 7 scale response ranging from never (1) to always (7). |
| Social Engagement Total | Assessed by the Australian Community Participation Questionnaire, the total is the sum of the 15 item short form questionnaire. A final score ranges from 0 – 7 with a higher score representing more social engagement. |

**Supplementary Table 2. Median Service Hours (IQR) used per week for each service type cluster**

|  | Cluster 1: Day Centre | Cluster 2: Social Support | Cluster 3: Outings |
| --- | --- | --- | --- |
| N people in each cluster | 844 | 151 | 40 |
| Day Centre | 4.0 (0-4.4) | 0 (0) | 1.8 (0-8.8) |
| Meal Services | 0 (0) | 0 (0) | 0 (0) |
| Outings | 0 (0) | 0 (0) | 9.6 (0-4.5) |
| Personal Care | 0 (0) | 0 (0) | 0 (0-5) |
| Respite Care | 0 (0) | 0 (0) | 0 (0) |
| Shopping | 0 (0) | 0 (0-1.5) | 0 (0) |
| Social Support | 0 (0) | 1.4 (0-2.0) | 0 (0-2.5) |
| Transport | 0 (0) | 0 (0) | 0 (0-1.5) |
| N service types used | 1 (1-1) | 2 (1-3) | 2 (2-4.75) |
